# Prenatal counseling for cloaca and cloacal exstrophy—challenges faced by pediatric surgeons

**DOI:** 10.1007/s00383-012-3133-3

**Published:** 2012-08-10

**Authors:** Andrea Bischoff, Maria A. Calvo-Garcia, Naira Baregamian, Marc A. Levitt, Foong-Yen Lim, Jennifer Hall, Alberto Peña

**Affiliations:** 1 Colorectal Center for Children, Division of Pediatric Surgery, Cincinnati Children’s Hospital Medical Center, 3333 Burnet Avenue, ML 2023, Cincinnati, OH 45229 USA; 2 Department of Radiology, Cincinnati Children’s Hospital Medical Center, Cincinnati, OH USA; 3 Fetal Care Center, Division of Pediatric Surgery, Cincinnati Children’s Hospital Medical Center, Cincinnati, OH USA

**Keywords:** Prenatal diagnosis, Anorectal malformation, Cloaca, Cloacal exstrophy, Covered exstrophy, Posterior cloaca

## Abstract

**Introduction:**

With the advance of prenatal imaging, more often pediatric surgeons are called for prenatal counseling in suspected cases of cloaca or cloacal exstrophy. This presents new challenges for pediatric surgeons since no specific guidelines have been established so far. The purpose of this review is to analyze our experience in prenatally diagnosed cloaca or cloacal exstrophy and to provide some guidelines for prenatal counseling of these complex congenital anomalies.

**Methods:**

A retrospective review of the medical charts of patients with prenatally diagnosed cloaca and cloacal exstrophy who received postnatal care in our institution between July 2005 and March 2012 was performed. Representative images of prenatal studies were selected from 13 cases to illustrate different scenarios and the recommendations given. In addition, a review of the literature was performed to support our advice to parents.

**Results:**

Eleven patients were female and two patients were male. The postnatal diagnoses were cloacal exstrophy (6), cloaca (5), posterior cloaca variant (1), and covered cloacal exstrophy (1). The selected abnormal prenatal imaging findings in these 13 patients included hydronephrosis (12), neural tube defect (8), omphalocele (7), lack of meconium at expected rectal location (7), vertebral anomaly (7), non-visualize bladder (5), distended bladder (5), hydrocolpos (4), dilated or echogenic bowel (3), umbilical cord cyst (3), separated pubic bones (2), and the “elephant trunk” sign (2). The prenatal diagnosis was correct in 10 cases, partially correct in two cases, and it was missed in one case. All parents received prenatal counseling depending on the specific diagnosis.

**Conclusion:**

The continuous technologic innovations in prenatal imaging make it possible to prenatally diagnose more complex anomalies including cloaca and cloacal exstrophy with increased levels of confidence and enhance the benefit of prenatal counseling. Together, these allow the parents to be better prepared for the condition and the care team to provide the best possible initial management in order to improve the outcomes of these challenging patients.

## Introduction

With advances made in prenatal imaging, more often pediatric surgeons are called for prenatal counseling in suspected cases of cloaca or cloacal exstrophy [1, 2]. This presents new challenges for pediatric surgeons since no specific guidelines for counseling have been established so far.

When a congenital anomaly is suspected prenatally, parents often become anxious, and they seek for all possible information related to the future of their unborn child, in hope to obtain the best care at birth that will result in the best possible outcome for their child.

The advantages of prenatal diagnosis include determining the viability of the fetus, offering the option of terminating pregnancy, and attempting to establish the functional prognosis for bowel, urinary and sexual function. All of these factors contribute to adjusting the parents’ expectations. In addition, it helps to elaborate on a management plan immediately after delivery, which determines the type of institution capable of providing proper care.

Until now the accurate prenatal diagnosis of a cloacal malformation remains challenging with the majority of reports in isolated series indicating that the diagnoses were limited to the more complex type of cloacas [3–6]. The first radiologic criteria for the prenatal diagnosis of cloacal exstrophy was proposed in 1995 [7] and was subsequently refined by others [8–12].

The purpose of this review is to analyze our own experience in a few cases of prenatally diagnosed cloaca or cloacal exstrophy in an attempt to provide some guidelines for prenatal counseling of these complex congenital anomalies.

## Methods

A retrospective review of the medical charts of patients with prenatally diagnosed cloaca and cloacal exstrophy who received postnatal care in our institution between July 2005 and March 2012 was performed. IRB approval was obtained for this study (IRB# 2012-1567).

Representative fetal magnetic resonance images (MRI) were selected to illustrate different scenarios and the recommendations given, based on our presumptive prenatal diagnosis and our previous experience in the management of those malformations. For counseling purposes, patients were grouped based on two diagnoses: (1) cloacal exstrophy and (2) cloaca. Additionally, specific advice was given in cases of neural tube defects (spina bifida, meningocele, myelomeningocele, and tethered cord) and severe hydronephrosis with significant kidney damage.

A review of the literature was performed to support our advice to parents.

## Results

A total of 13 patients were identified, 11 were female and 2 were male.

The average gestational age at the time of the fetal MRI was 23 weeks (range 18–31 weeks).

**Fig. 1:**
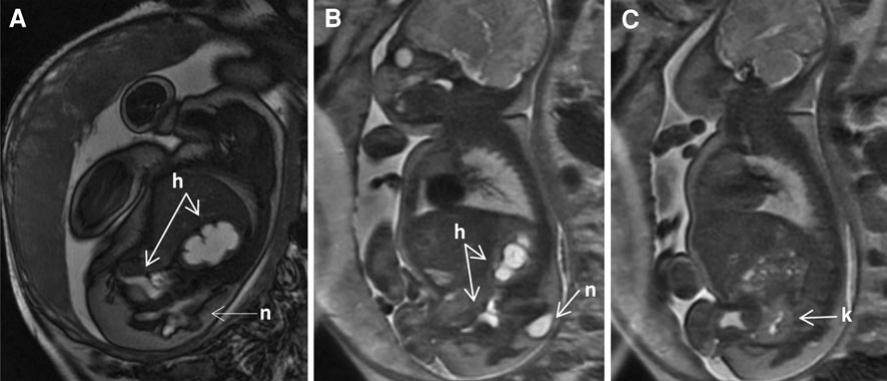
Prenatal MRI of a patient with cloacal exstrophy showing severe hydronephrosis and hydroureter (*h*) (**a**), neural tube defect (*n*) (**a**, **b**), and a pelvic kidney = k (**c**)

**Fig. 2:**
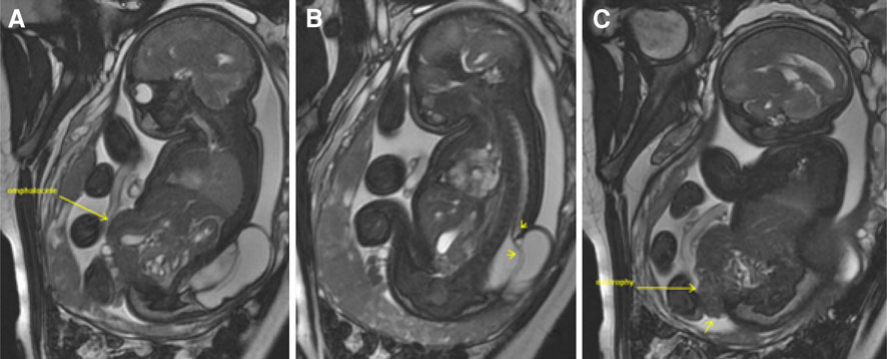
Prenatal MRI of a patient with cloacal exstrophy showing the omphalocele (**a**), neural tube defect (**b**) and bladder exstrophy (**c**)

**Fig. 3:**
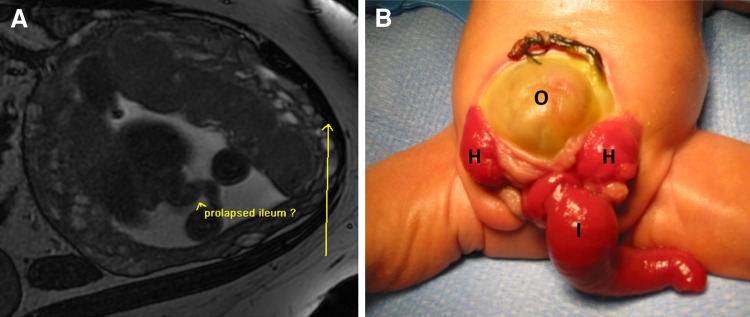
Patient with cloacal exstrophy showing the elephant trunk sign representing the intussuscepted ileum. **a** Prenatal MRI. **b** Postnatal image (*o* omphalocele, *h* hemibladders, *i* “elephant trunk” ileum)

**Fig. 4:**
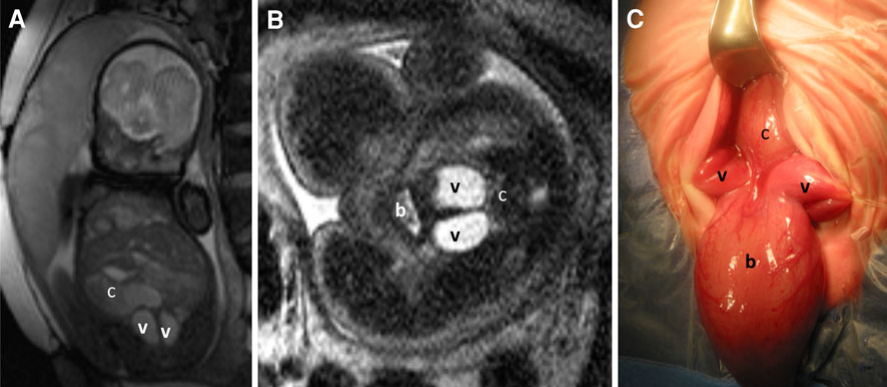
Prenatal MRI of a patient with cloaca. **a** Coronal view showing bilateral hydrocolpos (*c* colon, *v* vagina). **b** Axial view (*b* bladder, *v* vagina, *c* colon with calcification). **c** Operative picture (*b* bladder, *v* vagina, *c* colon)

**Fig. 5:**
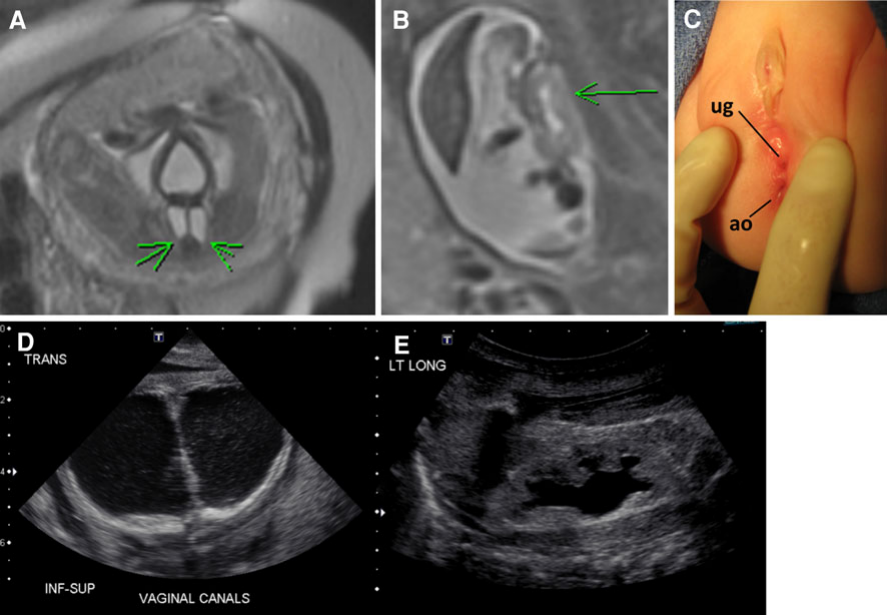
Prenatal and postnatal images of distended hemivaginas (hydrocolpos) and hydronephrosis in a patient with posterior cloaca [29]. **a** Bladder anterior and *arrows* pointing to distended hemivaginas. **b** Hydronephrosis. **c** Postnatal picture showing two perineal orifices (posterior located urogenital sinus = ug and anal opening = ao). **d** Postnatal ultrasound showing bilateral hydrocolpos. **e** Hydronephrosis

**Fig. 6:**
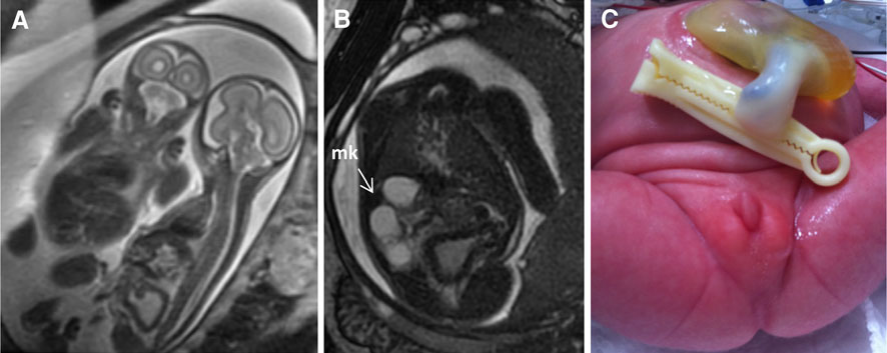
Fetal MRI of monochorionic, monoamniotic twins showing affected twin on the *right* with distended thick bladder (**a**, **b**), multicystic kidney = mk (**b**), and postnatal picture showing imperforate anus and umbilical cord cyst (**c**)

**Fig. 7:**
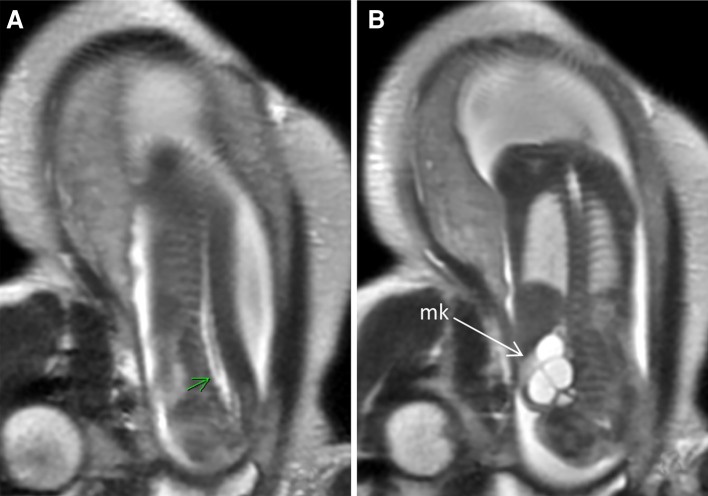
Prenatal MRI of a patient with cloaca. **a** *Arrow* pointing low conus (tethered cord). **b** Multicystic kidney (mk)

The key abnormal prenatal imaging findings in these patients included hydronephrosis (12 patients, Figs. 1a, b, 5b, 6b, 7b), neural tube defect (8 patients, Figs. 1a, b, 2a, b, 7a), omphalocele (7 patients, Fig. 2a), lack of meconium at the expected rectal location (7 patients), vertebral anomaly (7 patients), non-visualize bladder (5 patients), distended bladder (5 patients, Figs. 5a, 6a, b), hydrocolpos (4 patients, Figs. 4a, b, 5a), dilated or echogenic bowel (3 patients, Fig. 4a, b), umbilical cord cyst (3 patients, Fig. 3c), separated pubic bones (2 patients), and the “elephant trunk” sign that represented the intussuscepted bowel in the middle of the hemibladders (2 patients, Fig. 3a).

The postnatal diagnoses included cloacal exstrophy (6 patients), cloaca (5 patients), posterior cloaca variant (1 patient), and covered cloacal exstrophy (1 patient).

The prenatal diagnosis was correct in 10 cases. Two patients had rare anomalies that were not precisely diagnosed prenatally—posterior cloaca variant (Fig. 5) and covered cloacal exstrophy, both prenatally diagnosed as cloaca. In one case, the diagnosis was mistaken for an unusual gastroschisis, made with prenatal ultrasound evaluation without MRI, and the postnatal evaluation revealed that the patient actually had a cloacal exstrophy.

Two sets of twins were included in this series. One set had one affected fetus with cloaca (Fig. 6a–c) and another healthy fetus. The affected twin with cloaca died postnatally due to kidney failure, prematurity, and abdominal wall defect (umbilical cord cyst, Fig. 6c) that made the performance of hemodialysis and peritoneal dialysis extremely difficult. Prenatally, comfort care was suggested and agreed by the parents. The other set was represented by a minimally conjoined twin with compromised umbilical blood flow. One twin was diagnosed with limb–body wall complex and a double-outlet right ventricle with anticipated in-utero fetal demise. After extensive counseling, the mother elected to proceed with separation of the twins with cord coagulation of the twin with anticipated in-utero demise to minimize the potential risk to the co-twin. The procedure restored normal umbilical Dopplers of the co-twin but the mother developed preterm labor and the co-twin was delivered at 30 weeks gestation. The twin was found to have a covered cloacal exstrophy. This patient died 3 months postnatally due to necrotizing enterocolitis and respiratory failure related to prematurity.

### Prenatal counseling

#### Five parents received prenatal counseling for cloacal exstrophy

Advice included planning the delivery at a tertiary center willing to provide a “unified approach” (pediatric surgeon, urologist, gynecologist, orthopedic surgeon, and neurosurgeon sharing a unified plan) to avoid errors in the initial management with serious late repercussions.

These parents were informed that cloacal exstrophy represents the most severe of the spectrum of anorectal malformations. They were counseled that the importance of a unified management plan at birth [13], in order to optimize the future outcome, cannot be overemphasized. It consists in assuring that the entire gastrointestinal tract is incorporated into the fecal stream creating a real end colostomy [14]. The omphalocele should be closed during the initial procedure if possible. The Mullerian structures in females must be inspected, and the two hemibladders must be approximated and sutured together in the midline.

A technical mistake frequently seen in the neonatal management of cloacal exstrophy is to perform an ileostomy [15–17] without separating the bladder from the distal bowel and/or sacrificing the distal bowel by resecting it completely. The distal bowel should be preserved, even when it is very small initially. Over time, when exposed to the fecal stream, the distal bowel tends to grow enough to absorb water, forms solid stool, and therefore gives the patient the opportunity to respond to our bowel management program [18–20] which allows for the patient to have a colonic pull-through and be clean in the underwear [14]. Leaving the distal bowel attached to the urinary tract will not only impede the growth of that bowel but may also provoke hyperchloremic acidosis resulting from the absorption of urine by the colonic mucosa.

The decision of what tissue should be used to reconstruct the urinary tract should be taken only after the prognosis for bowel management has been determined.

Parents were also encouraged to think about the future of their children in three areas: bowel control, urinary control, and sexual function. Indicators of poor prognosis for bowel control include a very short piece of colon or absent colon (which can only be determined after birth during the initial surgical exploration), presence of myelomeningocele and or a tethered cord (usually detected prenatally), and a poor sacrum. The majority of patients with cloacal exstrophy will not have bowel control [14, 17, 21]. However, as previously mentioned, if they have enough colon to absorb water and form solid stool, they can have a colonic pull-through and be clean with a bowel management program [14], which according to our patients gives them a better quality of life than a permanent stoma.

The presence of spina bifida or tethered cord is also a poor prognosis for urinary control; the majority of patients require further urological reconstruction consisting in a bladder augmentation and the creation of a one-way valve conduit to empty the bladder with intermittent catheterization (Mitrofanoff principle [22]).

Sexual function in females can be achieved. The majority of patients have two hemivaginas and hemiuteri that are approximated on their most distal portion, yet some patients require vaginal replacement. In male patient, sexual function remains a serious challenge [23].

The presence of a large, closed neural tube defect (Fig. 2a, b) leads us to recommend a cesarean section as a mode of delivery to avoid the possibility of rupture. In addition, we were able to inform the parents about the potential negative implications for urinary function, bowel control and motor problems of the lower extremities.

#### Seven parents received prenatal counseling for cloaca

Parents were explained that in patients born with a cloaca, the urethra, the vagina, and the colon are joined together forming a common channel that opens as a single orifice in the same location of a normal urethra. The length of the common channel is cystoscopically determined after birth and it correlates with the complexity of surgical reconstruction that will be required as well as the functional outcome [24]. The use of a rotational fluoroscopy with 3D reconstruction [25] prior to the main surgery provides a more accurate anatomical assessment.

Poor prognostic indicators for bowel control include an abnormal sacrum (short) and presence of a tethered cord. Factors for poor prognosis for urinary control and need for intermittent catheterization include a long common channel and the presence of a tethered cord. Sexual function can be achieved.

In cases of cloacas with hydrocolpos, parents were advised to deliver in a tertiary center. They were made aware of the need of early vaginal decompression at the time of the colostomy opening and keeping the hydrocolpos drained until the definitive surgical repair. A frequent mistake during the newborn management of cloacas is not to drain the hydrocolpos [26], which can result in compression of the trigone producing an extrinsic uretero-vesical obstruction with resultant megaureters and hydronephrosis. In addition, the lack of drainage may lead to pyocolpos and rupture of the vagina. Approximately 60% of the cloacal patients have two hemivaginas and hemiuteri; in order to drain both hydrocolpos with a single tube, a window is created in the vaginal septum [26].

The colostomy must be made with two separated stomas to avoid fecal contamination of the urinary tract. The stoma must be created in the descending colon in order to avoid prolapse and assure that there is enough distal bowel for the future colonic pull-through [27].

## Discussion

Current prenatal imaging techniques (mainly MRI) allow for the identification of anatomic features that together, with a good index of suspicion, can establish a fairly accurate diagnosis of cloaca and cloacal exstrophy. There are, however, rare anatomic variants (such as posterior cloaca and covered exstrophies) that may escape prenatal detection.

In this series, three patients had a different postnatal diagnosis that did not prevent an adequate prenatal counseling. The first one represents the only case in which only ultrasound was utilized. On the report, the diagnosis of an unusual gastroschisis was made but it was mentioned that the body habitus of the mother made the exam inadequate and thus an MRI was indicated for further evaluation, but was never performed. We believe that if this patient had a fetal MRI, the diagnosis of a cloacal exstrophy would be more likely to have been made [28]. In the second case, an unusual type of cloaca described as posterior cloaca variant [29] was detected postnatally. In this anomaly, the urogenital sinus is posteriorly deviated and it opens either in the anterior rectal wall or very close to the anus (Fig. 5c), so it is easy to understand how it would be difficult to be differentiated from a classic cloaca as the difference relies in the location of the most distal portion of the urogenital sinus. Covered cloacal exstrophy [12, 21, 30] represents a spectrum of the cloacal exstrophy with the majority of cases represented by an intact abdominal wall or a small omphalocele, and low implantation of the umbilical cord. Yet, the intra-abdominal findings are consistent with those seen in cases of cloacal exstrophy (separated pubic bones, single perineal orifice, absent bladder neck, normal colon, short colon or almost absent colon, and duplicated appendix, etc). It is also easy to understand how the prenatal diagnosis is challenging in these cases since it is a rare anomaly with many anatomic variants.

The main challenge faced by pediatric surgeons while counseling parents is the capacity to make an accurate prenatal diagnosis due to good imaging evaluation but without the full elements that are needed to predict the functional outcome. Another challenge is that more often the prenatal diagnosis is possible in patients with more complex defects and associated malformations that usually reflect a guarded prognosis for bowel and urinary control. Nevertheless, it is advantageous to provide counseling to parents concerning the need to deliver at a tertiary center with experience in managing these malformations, in order to avoid errors during the newborn management that could have a negative impact on the future functional prognosis of their children.

Persistent cloaca in one of the fetuses in a set of monozygotic twins is a rare occurrence [31]. In singleton pregnancy complicated by severe oligohydramnios or anhydramnios due to dysplastic renal disease, there is increased risk of neonatal death from pulmonary hypoplasia [32]. In our set of twins, the pulmonary hypoplasia was avoided by the amniotic fluid production of the normal twin. Prenatal counseling therefore had to take into consideration the risk of prematurity in the set of twins, the risk of in-utero fetal demise of the affected twin that ultimately could affect the normal twin, the feasibility of dialysis techniques for premature babies (low birth weight) with abdominal wall defects and in need of abdominal surgery. Hemodialysis is challenging in newborns and peritoneal dialysis is usually the preferred method. But in our patient a colostomy, a vesicostomy, and a closure of the abdominal wall defect would have to be performed soon after birth. The underlying defects as well as the surgical procedures required would delay the commencement of peritoneal dialysis or render it impossible. Taking all of these factors into consideration, comfort care was suggested during prenatal counseling and agreed by the parents.

The second set of twins belonged to the cloacal exstrophy group and there is a theory of twinning as an etiology of cloacal exstrophy [33–35].

## Conclusion

The continuous technologic innovations in prenatal imaging make it possible to prenatally diagnose more complex anomalies including cloaca and cloacal exstrophy with increased levels of confidence and enhance the benefit of prenatal counseling. Together, these allow the parents to be better prepared for the condition and the care team to provide the best possible initial management in order to improve the outcomes of these challenging patients.
